# Lessons from One Fastidious Bacterium to Another: What Can We Learn about *Liberibacter* Species from *Xylella fastidiosa*

**DOI:** 10.3390/insects10090300

**Published:** 2019-09-16

**Authors:** Angela Kruse, Laura A. Fleites, Michelle Heck

**Affiliations:** 1 Department of Plant Pathology and Plant-Microbe Biology, Cornell University, Ithaca, NY 14853, USA; 2 Boyce Thomson Institute, Ithaca, NY 14853, USA; 3 Emerging Pests and Pathogens Research Unit, Robert W. Holley Center, United States Department of Agriculture Agricultural Research Service (USDA ARS), Ithaca, NY 14853, USA

**Keywords:** Huanglongbing, *Candidatus* Liberibacter asiaticus, *Diaphorina citri*, citrus greening, *Xylella fastidiosa*, *Homalodisca vitripennis*, Pierce’s disease, plant pathology, vector biology, bacteriology, bacterial pathogen transmission, hemiptera, biological control

## Abstract

Huanglongbing is causing economic devastation to the citrus industry in Florida, and threatens the industry everywhere the bacterial pathogens in the *Candidatus* Liberibacter genus and their insect vectors are found. Bacteria in the genus cannot be cultured and no durable strategy is available for growers to control plant infection or pathogen transmission. However, scientists and grape growers were once in a comparable situation after the emergence of Pierce’s disease, which is caused by *Xylella fastidiosa* and spread by its hemipteran insect vector. Proactive quarantine and vector control measures coupled with interdisciplinary data-driven science established control of this devastating disease and pushed the frontiers of knowledge in the plant pathology and vector biology fields. Our review highlights the successful strategies used to understand and control *X. fastidiosa* and their potential applicability to the liberibacters associated with citrus greening, with a focus on the interactions between bacterial pathogen and insect vector. By placing the study of *Candidatus* Liberibacter spp. within the current and historical context of another fastidious emergent plant pathogen, future basic and applied research to develop control strategies can be prioritized.

## 1. Introduction and Historical Context

Huanglongbing (HLB) is the most serious disease of citrus and is causing economic devastation in Florida. The first probable record of HLB was 260 years ago in India, where growers reported citrus ‘dieback’ in the 18th century [1]. In the 1960s, HLB was thought to be caused by *Citrus tristeza virus* [2]. HLB has since been associated with ‘*Candidatus* Liberibacter africanus’ (*C*. Laf) in Africa and some parts of Asia, ‘*Candidatus* Liberibacter americanus’ (*C*. Lam) in South America, and ‘*Candidatus* Liberibacter asiaticus’ (*C*. Las) in the United States, Brazil, and Asia [3,4,5,6,7,8,9,10,11,12]. *C*. Las is the most aggressive, widespread, and most studied of these pathogens [13]. *C.* Las is transmitted between plants by *Diaphorina citri* (*D. citri*), also known as the Asian citrus psyllid. *D. citri* was detected in Florida in 1998, and is now understood to invade groves from many kilometers away, depending on the grove landscape [14,15]. HLB was detected in 2005, and the pathogen subsequently spread to every citrus-producing county in that state [13]. Extensive efforts are being made to understand this disease, but no control strategy has been effective. *C.* Las cannot currently be grown in pure culture. Prominent researchers have stressed the importance of unconventional thought and innovative solutions to address this problem [16]. Although this disease may appear to be insurmountable, there was a time when the grape industry was equally desperate for solutions to manage Pierce’s disease. 

Pierce’s disease was first reported in grapevine in 1892 [17]. The causative agent was originally thought to be a virus, but was later proven to be the bacterium *Xylella fastidiosa,* which was later also associated with citrus variegated chlorosis [17,18]. At the time, *X. fastidiosa* was unculturable. Pierce’s disease reached an epidemic status from 1930 to 1940, and increased scientific efforts led to the identification of insect vectors capable of transmitting the bacterium. Initially, the primary vector was identified as the blue-green sharpshooter (*Graphocephala atropunctata*) [19]. Later, the invasive glassy-winged sharpshooter (GWSS; *Homalodisca vitripennis*) worsened the disease situation by transmitting the pathogen more widely [19]. *X. fastidiosa* was successfully cultured in 1978, leading to a quantum leap in understanding of the pathogen and its transmission [20]. Broadly, each step forward in understanding of the biology of *X. fastidiosa* has resulted in a paradigm shift in our understanding of this pathosystem and plant pathology as a whole (reviewed in [21]). *X. fastidiosa* has since been observed to cause disease in a wide range of plant hosts [18,19,22,23,24]. 

The deluge of information about the pathogen, insect vector, and host tolerances was successfully leveraged for control of Pierce’s disease in grape using a multi-pronged management strategy [25]. A major component of this strategy focused on control of the GWSS using containment, detection, rapid response, and outreach [25]. Containment involved the regulation of nursery stock and bulk grape shipments from infected areas, certification programs and the removal of infected vines. Detection primarily relied on monitoring the presence of GWSS via sticky traps. Rapid response was taken after detection of Pierce’s disease or the GWSS and involved visual surveys and additional monitoring. Outreach improved the compliance with the aforementioned efforts among growers and the public via educational efforts. In tandem with control of the GWSS, conventional breeding and genetic engineering produced grapevines with increased resistance to Pierce’s disease [25,26]. Pierce’s disease is now well managed in California, to the credit of this holistic management plan. Meanwhile, continual molecular and breeding efforts are ongoing for even more durable solutions without spending valuable time responding to a disease crisis. 

*X. fastidiosa* and *C.* Las have important differences, such as their host range and vascular habitation. However, management strategies for xylem and phloem limited pathogens primarily involve the control of the insect vector and the development of resistant host plants [27,28], strategies which may be broadly useful for management of plant pathogens with vascular tropism [29]. *X. fastidiosa* and *C.* Las also have important similarities, such as their ability to survive in both a plant host and a hemipteran insect vector, and reduced genomes lacking type III secretion systems (Table 1). This review focuses on the key data and experimental workflows that led to our current understanding and control of Pierce’s disease, and how these approaches can be applied to the study and eventual control of HLB.

## 2. Pathogen

### 2.1. Background and genomic resources

*X. fastidiosa* was the first plant pathogen sequenced (Table 2) [30]. Its genome is approximately 2.7 megabases in size, with a 52.7% GC content, showing a significant reduction in size compared to other sequenced Xanthamonads, such as *Xanthamonas campestris* pv. campestris strain 17, which has a genome size of approximately 5 megabases [30]. Comparative genomics of *Xylella fastidiosa* strains Temecula and 9a5c, which cause Pierce’s disease and citrus variegated chlorosis, respectively, revealed that these strains share 98% of the same genes, with differences in genomic islands resulting from phage-associated chromosome rearrangement and deletions [31]. Additionally, comparative genome analyses of *X. fastidiosa* strain EB92, which colonizes grapevines but does not cause disease, with pathogenic strain Temecula1 revealed that only 11 genes were unique to Temecula1 [32]. This indicates that a small number of genes can alter the pathogenicity, host specificity and resulting disease phenology of *X. fastidiosa*. 

There are currently five complete genome sequences of *C*. Las deposited in GenBank, with several more that are not yet fully assembled [34,35,36,37]. *C.* Las has a highly reduced genome of approximately 1.23 megabases, with a low GC content of 36.5% [37]. Important for comparison, the genomes of *C*. Lam [38], *C.* Laf [39], and the causative agent of zebra chip disease, *Candidatus* Liberibacter solenacearum (*C*. Lso) have been published [40]. *C.* Las currently cannot be cultured, though studies have prolonged its viability *in vitro* [77,78]. A comprehensive review of efforts to culture *C.* Las can be found in [79]. In a strategy mirroring that taken from *X. fastidiosa*, comparative genomics was used to determine the key genomic regions that may dictate host and vector specificity [34,41,42,43,44]. The inability to culture any of the pathogenic Liberibacter species precludes validation of any candidate genes involved in citrus or psyllid colonization. *C.* Las may have at least two prophages within its genome, and rearrangements within prophage regions have been shown to result in genomic variants [80]. The prophage variants present in *C.* Las populations vary between bacteria isolated from *D. citri*, citrus plants, and even another phytophagous hemipteran insect (*Ferrisia virgata*) [80,81]. 

### 2.2. Virulence

The genome sequence of *X. fastidiosa* revealed that the pathogen lacks a type III secretion system [23,82], but does possess a type I secretion system along with annotated type I effectors including hemolysins and bacteriocins [30]. Knockout of *tolC*, the outer membrane component of the type I secretion system, resulted in avirulence and hypersensitivity to phytoalexins in *X. fastidiosa* [83]. *X. fastidiosa* has only one copy of each gene forming the type I secretion system, including *tolC* (Table 3) [30]. Single-copy genes are important targets to control bacterial virulence. The type II secretion is also known to affect virulence [84], and *X. fastidiosa* and other pathogenic members of *Xanthomonadaceae* have very similar type II secretion systems. Screening of *X. fastidiosa* mutants also revealed a diffusible signaling factor (*Xf*DSF) that is required for virulence and encoded by genes in the *rpf* gene cluster [85]. Expression of *rpfF* induces *Xf*DSF in grapevines and reduced *X. fastidiosa* spread within the plant relative to a near isogenic line that carried a non-functional form of the gene [86,87]. This study broadly shows that perturbance of cell-to-cell communication may be an effective bacterial control strategy. Proteomics was used to analyze the *X. fastidiosa* secretome, and found that a lipase/esterase (LesA) was abundant in secretome and in outer membrane vesicles [88]. It is orthologous to a cell wall degrading enzyme from another *Xanthomonas* species, and is a key pathogenicity factor for *X. fastidiosa*. 

The genome of *C*. Las also shows the lack of a type III secretion system, and only the inner membrane component of the type II secretion system [34]. The bacterium has 14 identified ABC transporters which form the inner membrane component of the type I secretion system, but only one copy of *tolC* [34,89]. Analogous to *X. fastidiosa*, *tolC* may be a promising target for disease control. *C*. Las also has two novel type V autotransporters, dubbed LasAI and LasAII, that may target plant mitochondria [90]. *C.* Las lacks an Rpf cell-cell communication system, and it is not known what, if any, diffusible factors may trigger quorum sensing and how that may relate to virulence. Correlations have been documented among the relative abundances of the *D. citri* bacterial endosymbionts and *C.* Las in tissues where they co-localize, strongly suggesting the existence of an interspecies interaction using an undescribed quorum sensing mechanism [66,91,92]. Future research should focus on understanding virulence and quorum-sensing factors that may contribute to *C.* Las life traits and pathogenicity in the plant and insect vector. Quorum sensing is further discussed below in the section describing biofilm formation.

An essential component of bacterial virulence is avoidance of the host immune system. Lipolysaccharides (LPS) are a principal component of the outer membrane (OM) of most gram-negative bacteria. This complex molecule, which imparts structural stability to the cell, consists of three parts: lipid A, which constitutes the bulk of the OM outer leaflet, core oligosaccharides, and a terminal o-antigen polysaccharide chain. The lipid A component of LPS is a well-studied elicitor of the defense response in both plant and animal systems, and although both *X. fastidiosa* and *C*. Las express LPS, the bacteria are able to evade initial detection and establish infections in their respective hosts. Recent research on *X. fastidiosa* strain Temecula1 demonstrates that an unusually long terminal o-antigen polysaccharide chain functionally shields the inner lipid A component of LPS from detection by the plant host [93]. *C*. Las may adapt a similar strategy in *D. citri,* where the expression of several genes involved in LPS biosynthesis are down-regulated relative to *in planta* [94]. Interestingly, *C*. Las relative *C*. Lam has shed most pathogen associated molecular patterns (PAMPs), and appears to avoid LPS biosynthesis altogether [38].

Though many studies in *X. fastidiosa* rely on mutagenesis, which is not yet possible with *C*. Las, they have underscored the importance of secretion system components and mobile signaling elements in virulence and provide potential targets for silencing which may be essential in *C.* Las virulence. As the closest culturable relative of *C.* Las, *Liberibacter crescens* is an important genetic resource to understand *Liberibacter* genetics and the core genes necessary for virulence and culturability. A previous study used Tn*5* mutagenesis to identify 314 genes necessary to culture *L. crescens* [95]. Of these essential genes, 76 of them are absent in the pathogenic, unculturable *Liberibacter* species including *C.* Las. These data provide possible molecules essential for the culture of *C.* Las, as well as genes shared between the two species that may be essential for *C.* Las pathogenicity, and therefore are promising targets for inhibition [95]. The above work illustrates the utility of *L. cresecens* as a model to study *C.* Las. This principle has been expanded in a recent study, which shows that *L. crescens* provides a platform to study *C.* Las biofilm formation [96]. Current and future studies can leverage *L. crescens* as a tool to study *C.* Las virulence and other important bacterial phenotypes *in lieu* of culturability of the pathogen. 

### 2.3. Biofilm formation

Biofilm formation is a crucial step in the lifecycle of pathogenic bacteria, and its disruption can have enormous effects on disease outcome [97]. *X. fastidiosa* can form biofilms in both the insect vector and its plant hosts. As discussed earlier, the *rpf* gene cluster controls the synthesis and recognition of *Xf*DSF, which is essential for biofilm formation in both the plant and insect [98]. Interestingly, the *X. fastidiosa* biofilms have a different appearance and morphology in the insect and the plant, indicating that environmentally-dependent gene expression is responsible for biofilm morphology [98]. The *X. fastidiosa* genome revealed an array of fimbrial and non-fimbrial adhesins, which were shown to be involved in agglutination, attachment to host cells, and pathogenicity [99,100,101,102]. The role of four *X. fastidiosa* adhesion proteins (PilA2, PilC type 4 pili proteins, and XadA1, XadA2 afimbrial adhesins) in biofilm formation was analyzed, and the proteins are expressed differentially during the stages of biofilm formation [103]. These proteins were present in the xylem vessels of the plant during infection. They were also expressed at differing time-points during infection [103]. Deletion of the outer membrane protein MopB in *X. fastidiosa* affects biofilm formation and virulence. In addition, deletion of MopB completely eliminated twitching motility, a key process that is intimately tied to biofilm formation and is required for systemic colonization of the plant xylem [104,105]. 

Quorum sensing is a chemical communication mechanism that bacterial populations use to orchestrate motility, biofilm formation and virulence. In most bacteria, quorum sensing mechanisms consists of a regulatory network involving two genes: *luxI* and *luxR*. The former gene type encodes enzymes that produce a variety of chemically distinct acyl-homoserine lactone (AHL) quorum sensing molecules. When the concentration of AHL molecules reach quorum levels, they activate *luxR* genes, which are AHL-responsive transcriptional regulatory genes [106]. Intriguingly, no *luxI* gene orthologs are found in the *C*. Las genome, yet *C*. Las has two functional *luxR* genes that are expressed during plant and insect infection [34]. These transcription factors are upregulated when the bacterium is in the psyllid and bind to the promoter of a *C*. Las gene involved in the production of type IV tight adherence-pili (tad, [107]), suggesting a role for the tad pilus in psyllid colonization. 

Interactions between *C.* Las and other bacterial species may enable *C*. Las to form biofilms without a functional *luxI* gene. *C*. Las is purported to form biofilms in *D. citri* guts, where it replicates to high levels, but has not been observed in a biofilm within the plant [108,109,110]. The bacterium has a *luxR* but not a *luxI* gene, indicating that it can sense AHLs to induce biofilm formation, but cannot synthesize them independently [34]. It is possible that *C*. Las perceives AHLs from *D. citri* endosymbionts, the plant host, or *D. citri* itself. This hypothesis is supported by the fact that ‘solo’ LuxR proteins have been observed to perceive AHLs from plants, other bacteria, or even exogenous applications [111]. The titers of endosymbionts are also positively correlated with that of *C.* Las, supporting a role for positive regulation between *C.* Las and other bacterial species [92]. *Wolbachia* and *C.* Las also co-localize within the same gut cells, and their physical proximity may be indicative of cooperation between the two species and interspecies signaling [58,112,113]. 

### 2.4. Biocontrol 

There are many possible methods for biological control of Pierce’s disease. Naturally occurring, avirulent strains of *X. fastidiosa* can be used as biological control in vineyards [114]. Further characterization of *X. fastidiosa* biological control strain EB92-1 demonstrated that this strain lacks 10 putative pathogenicity factors and infects grapevine but does not cause disease [32]. The endophytic bacterium *Paraburkholderia phytofirmans* strain PsJN can colonize grapevine while restricting the growth of *X. fastidiosa* [115]. Furthermore, paratransgenesis approaches have shown promise for *X. fastidiosa* control, with one study inducing a genetically manipulated bacterium in the genus Alcaligenes to colonize the GWSS foregut and compete with *X. fastidiosa* [116]. *X. fastidiosa* has four predicted prophages within its genome, designated XfP1 through XfP4, which can be evaluated for control of the bacteria [30]. 

In parallel to the evaluation of biological control strains for *X. fastidiosa, L. crescens* can also be evaluated for biological control of *C.* Las, as it can be transformed and cultured. The genomes of some strains contain varying integrated prophages, and several groups are examining the possibility of employing phage therapy for disease control [117,118,119]. The most well studied strain of *C.* Las, psy62 has two prophages within its genome [117]. In addition, the psyllid endosymbiont *Wolbachia* encodes a repressor of a lytic phage gene promoter, and this protein is a candidate target for control of *C*. Las in the psyllid vector [113]. Induction of lytic prophages is a promising area for control of *C.* Las. 

### 2.5. Global Outlook 

Climate change is expected to have profound effects on the distribution of crops, plant pathogens, and insect vectors. This is particularly relevant in the case of *X. fastidiosa*, because cold curing is observed in grapes infected with Pierce’s disease [25]. That is, infected grapevines that experience cold temperatures can be cured of *X. fastidiosa.* This is thought to be the reason that Pierce’s disease is not found in grape-growing areas with colder winters such as New York, Washington, and Oregon. The severity of Pierce’s disease is negatively associated with severity of winter [120,121]. Modeling predicts that global warming will alter distribution of crops [122], and where winter temperatures have increased in recent years, Pierce’s disease distribution has increased as well [123]. These studies provide a strong case that climate change will increase the range of *X. fastidiosa* and its insect vector, exacerbating and spreading Pierce’s disease. 

*C.* Las is exceptionally heat tolerant relative to the other pathogenic Liberibacter species. Citrus infected with *C.* Las has been shown to maintain high titers of *C.* Las in very warm conditions (cycles of six hours at 35° C and nine hours at 24 °C for 90 days) [124]. However, heat treatment of *C.* Las-infected citrus at 40–42 °C for at least 48 hours reduces bacterial titer [125]. Taken together, these studies indicate that the threshold for *C.* Las heat susceptibility is between 35 °C and 40 °C, likely depending on duration of heat exposure. In contrast, *C.* Lam has been shown to be heat sensitive: titers are significantly diminished at a moderate temperature regime (cycles of six hours at 32 °C and nine hours at 24 °C for 90 days) [124]. Similar observations of heat sensitivity have been reported for *C.* Laf [126]. These observations suggest that *C.* Las will be able to tolerate increasing global temperatures, and could outcompete other Liberibacter species and expand in range as temperatures rise. The HLB field can learn from the unexpected climate-driven emergence of *H. vitripennis* as a *X. fastidiosa* vector by monitoring and predicting the geographical range of psyllid species over time.

## 3. Vector

### 3.1. Path through vector

Insect transmission is a crucial step that allowed both *X. fastidiosa* and *C.* Las to establish disease epidemics. Understanding the relationship between bacteria and insect vector is a high priority, and control of the insect vector is the most important factor for disease control [25]. *X. fastidiosa* and *C.* Las take very different paths through their respective insect vectors. *X. fastidiosa* is ingested by *H. vitripennis* while the insect uses its piercing-sucking stylet to feed on plant xylem. The bacteria are acquired into and replicate in the insect foregut, but do not cross the gut barrier to circulate within the insect’s other organs [127]. This transmission process is defined as propagative and foregut-borne. *C.* Las takes a longer path through its insect vector. *D. citri* feeds on citrus phloem using its piercing-sucking stylet, and ingests *C.* Las. *C.* Las moves into the insect gut, where it is acquired and replicates in cells in the midgut [112,128]. It exits the gut cells to circulate in the hemolymph until it reaches and replicates in the salivary glands to be inoculated into the next plant host [98]. 

### 3.2. OMICs Resources

Genomic and other OMICs resources can greatly accelerate this research. The genome of *H. vitripennis* is available as part of the i5K pilot program [45]. A *de novo* transcriptome and mRNA profile are also available for *H. vitripennis* [55,56]. These datasets are expected to expedite future research and provide novel insect targets for control. Websites such as https://nature.berkeley.edu/xylella/ provide information about Pierce’s disease biology and management, although, to the best of our knowledge, there is not a central repository for bioinformatic resources and OMICs data for the field of Pierce’s disease. 

The *D. citri* genome was sequenced, and annotation efforts have improved its quality [46,47,48]. The *D. citri* whole-body, antenna, abdominal, and gut transcriptome have been published [57,58,59]. The whole-body proteome, gut proteome, and hemolymph proteome are additional resources to identify transmission targets [58,65,66]. Interestingly, there are many more OMIC resources for *D. citri* than *H. vitripennis*, likely due to the unculturable nature of the pathogen and the expansive funding efforts that have been directed to discover solutions to citrus greening [129]. These are expected to compensate for this intractability by providing a large volume of potential genes or proteins from *D. citri*, *C.* Las, and *D. citri* bacterial endosymbionts that can be targeted using RNA interference or other inhibition strategies *in lieu* of mutant screens [130,131]. The website https://citrusgreening.org/ provides a central repository for resources and information pertinent to citrus greening, and this website could be further leveraged as a tool for the HLB research community to share OMICs data, thus avoiding duplication of efforts. 

### 3.3. Transmission determinants

Most potential transmission-reducing strategies that are being evaluated for Pierce’s disease resulted from mutant screens in *X. fastidiosa.* For example, in one study, mutant strains of *X. fastidiosa* were screened to identify those deficient in attachment to polysaccharides, and by extension, adhesion to insect foregut cuticles [132]. This study implicated hemagglutinin-like proteins in adherence to the insect vector, and mutants in hemagglutinin proteins were indeed less transmissible [132]. N-acetylglucosamine inhibited bacterial adhesion to vector foregut extracts and intact wings [132]. Lectins such as wheat germ agglutinin, monomeric and multimeric forms of N-acetylglucosamine, antibodies to whole bacterial cells, extracellular polysaccharides, and afimbrial adhesins all negatively impacted transmission [101]. Another study evaluated *X. fastidiosa* mutants’ ability to be transmitted at various time points [102]. Mutants of fimbrial and afimbrial adhesins were deficient in adhesion to vector gut, and regulatory mutants (*rpfF* controlled) were deficient in initial adhesion and retention [102]. This study provided a molecular timeline showing the most important bacterial factors at each stage of transmission, which can broadly be applied to *C.* Las transmission. 

*D. citri* is a genetically heterogeneous, sexually reproducing species, and the genetic background of individual insects can influence their interactions with *C.* Las. A recent study showed that *D. citri* color morphology impacts its vectoring capacity [133]. *D. citri* can be found in three color morphs: blue, yellow, and gray. Blue individuals were found to harbor a lower titer of *C.* Las and the other bacterial endosymbionts, and transmit the pathogen less efficiently. A copper binding protein called hemocyanin, which is thought to be responsible for the insects’ blue color, may be responsible for these effects [133]. Vector competency has also been shown to vary naturally among *D. citri* populations, and to be heritable over many generations [134]. Additional studies showed that *C.* Las manipulates its insect vector to fly further and faster, and lay more eggs [135]. The molecular basis of this vector manipulation may be a promising area of study that can begin by mining the aforementioned datasets for proteins involved in this response. For example, increased egg production may be a result of the dramatic up-regulation in vitellogenin protein expression observed in *C.* Las-exposed hemolymph [66]. 

### 3.4. Feeding

Insect feeding is a promising target for transmission control. Preventing feeding is a major goal of insecticide and noninsecticide-based strategies for the control of both *H. vitripennis* and *D. citri*. White kaolin has been used to control GWSS preference for grapevine, and increases insect mortality [136,137]. Harpin, which elicits a plant immune response rather than directly attacking the insect vector and is thought to induce systemic acquired resistance [138,139], was evaluated in a field study and reduces Pierce’s Disease incidence [137]. Consistent with the non-circulative mode of *X. fastidiosa* transmission, time spent probing plants was found to be more important than ingestion time for inoculation of this pathogen into plants [140]. Transmission of *X. fastidiosa* can also be predicted using modeling, and shows that the number of insects feeding increases bacterial inoculation and leads to earlier onset of symptoms [141]. This is a likely commonality with *C.* Las transmission, as the pathogen inoculation efficiency greatly increases with number of insects feeding [112,142]. An early study also detected two unidentified proteins in the hemolymph, salivary sheath, and saliva of *H. vitripennis*. The authors speculate that this protein may be trafficked from the hemolymph to the salivary glands and subsequently the salivary sheath. Future research should focus on identifying these proteins via modern proteomics techniques and targeting them to inhibit *H. vitripennis* feeding [143]. 

**Figure 1:**
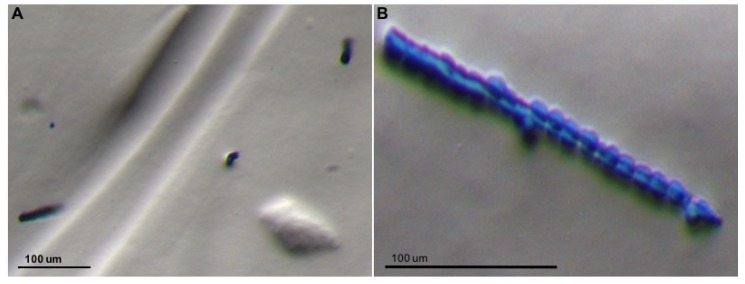
Stylet sheaths deposited by adult *D. citri* feeding on semi-solid agarose diets. *D. citri* stylet sheaths recovered from an artificial diet chamber containing five adult *D. citri*. Diets were removed from the chamber and post-stained with colloidal blue and imaged using a Leica M205 stereo microscope at low magnification (**A**) and higher magnification (**B**).

Several studies have focused on the feeding structures of *D. citri*, including the salivary glands, stylet, and stylet sheath. Structural studies are available for the stylet and stylet sheath of *D. citri* and the potato psyllid [144,145,146,147]. *D. citri* stylet sheath formation and morphology can be visualized independently of a plant using artificial diet systems (Figure 1), enabling research on the development of molecules that inhibit sheath formation, plant feeding and transmission. The *D. citri* secreted salivary proteome is also available and can be mined for proteins that may be involved in feeding [148]. A total of 89 proteins have been identified in soluble *D. citri* saliva, of which 86 were from *D. citri* and three were from its bacterial symbiont *Wolbachia* [148]. *D. citri* salivary proteins include enzymes (consisting of oxidoreducatases, proteases, phosphatases and kinases, and transferases), cytoskeletal proteins, sheath proteins, receptor proteins, transporter proteins, nucleic-acid binding proteins, other *D. citri* proteins, and endosymbiont proteins [148]. A structural sheath protein, which was described in aphid stylet sheaths [149], has yet to be identified for *D. citri* or other psyllids. Future research can focus on characterization and silencing of the genes encoding for *D. citri* salivary proteins for potential control of *D. citri* feeding and sheath formation. 

### 3.5. Biocontrol

Biological control of the vector is another approach to disease management. Examples include the use of predators and parasitoids of the insect vector and insect infecting fungi and viruses, which reduce the vector population. Biological control of the GWSS primarily relies upon release of parasitic wasps that attack the eggs of the insect. Several species within the genus *Cosmocomoidea* (previously *Gonatocerus*) are deployed against the GWSS, including C*. ashmeadi*, *C*. *morgani*, and *C. morrilli* [150]. Based on a California Department of Food and Agriculture (CDFA) report, 2.61 million biological control agents have been released in California from the start of the program in 2001 through 2017 [150]. In an analogous approach, parasitic wasps, including *Tamarixia radiata* and *Diaphorencyrtus aligarhensis*, are being deployed to manage *D. citri* [151,152,153]. Psyllid infecting viruses have been described and may be leveraged as biocontrol tools with additional research. In particular, a novel *Diaphorina citri-associated C virus* and a virus in the insect-infecting densovirus family have been associated with *D*. *citri* [154,155]. Psyllids infected with entomopathogenic fungi have been discovered and may be used together with novel lures and traps that may aid in fungal spore dissemination in a grove [156,157]. *Isaria fumosorosea* can be readily cultivated in laboratory conditions and causes *D. citri* mortality between 4.9 and 6.1 days after exposure [156]. *Hirsutella citriformis* has also been observed infecting adult *D. citri* in Florida citrus groves, where it causes mortality in an average of 23% of *D. citri* individuals [157]. However, care must be taken in a holistic biocontrol approach, as antagonistic interactions between entomopathogens and parasitoids used to control *D. citri* have been documented [158]. Research on augmentative strategies, in particular, molecular regulation of entomopathogen virulence in the psyllid, will be invaluable for such efforts to reduce vector and pathogen populations to levels that are inconsequential for transmission. 

## 4. Host

### 4.1. OMICs as Resources for Breeding

The abundance of OMICs studies provides detailed information about host factors contributing to *X. fastidiosa* infection or tolerance. A paired proteome, transcriptome, and metabolome of grapevine infected with *X. fastidiosa* showed an accumulation of gamma-aminobutyric acid and increased iron and copper chelating activity, as well as induction of pathogenesis-related proteins and phytoalexins [60]. This study also showed up-regulation of cell wall modifying proteins consistent with xylem wall thickening during infection [60]. Proteomic analysis of infected versus healthy grapevine at various time-points implicated thaumatin-like proteins, glycoprotein, and formate dehydrogenase in resistance to *X. fastidiosa*, while decreased expression of heat shock proteins was associated with susceptibility [67]. Proteomic comparison of tolerant versus susceptible grapevines identified β-1, 3-glucanase, peroxidase, and a subunit of oxygen-evolving enhancer protein 1 only in a tolerant variety, and found lower levels of free sugars and amino acids in tolerant varieties [68]. This study provides evidence that the nutrient composition of xylem sap can positively or negatively influence bacterial growth. This is consistent with the fact that susceptible grapevine xylem sap enhances biofilm formation in *X. fastidiosa in vitro* [60]. These datasets provide an enormous amount of information that can inform breeding efforts, for example, by identifying defense proteins or metabolic pathways associated with resistance. The use of OMICs datasets to inform functional studies and identify resistance factors is expected to be part of a long-term interdisciplinary effort to generate grapevine varieties resistant to *X. fastidiosa.* Development and testing of grapevine cultivars for resistance is underway, and in the meantime, Pierce’s disease is being controlled in California via multiple efforts [25]. 

Hosts of *C*. Las include every known species of citrus, with the species with least severe outcomes deemed *C*. Las ‘tolerant’. A metagenomics approach studied the microbial diversity of *C*. Las-infected citrus phloem. This confirmed the previously published *C.* Las genome, and also detected no other pathogen DNA, including viruses or viroids, implying possible competition between *C*. Las and other phloem-limited microbes, such as CTV and *Spiroplasma citri*, which is supported by a study funded by the California Citrus Research Board [49]. The authors estimate based on their data that the phloem sample contains 1.7 *C*. Las cells per phloem cell [159]. Time-course transcriptomics of ‘tolerant’ rough lemon and susceptible sweet orange in response to *C*. Las showed that more genes were differentially expressed in rough lemon at early time points, and fewer at late time points [61]. Phloem transport was much less affected in ‘tolerant’ rough lemon, and provided candidate genes for transformation and breeding of citrus [61,160]. Microarray analysis compared healthy and *C*. Las-exposed sweet orange, and also found gene categories such as sugar transport to be affected [62].

### 4.2. Transgenic Strategies

Several transgenic lines of grapevine have shown promise in reducing Pierce’s disease severity. A fusion protein was made between one protein that binds to the cell surface of *X. fastidiosa*, and one that penetrates the cell membrane and kills the bacteria. This fusion protein was expressed in plants and conferred increased resistance to Pierce’s disease [161]. *X. fastidiosa* mutants lacking a hemagglutinin-like *X. fastidiosa* (HxfA) protein are hypervirulent, indicating that this protein’s natural function is to suppress virulence traits [162]. Transgenic grapevines expressing HxfA had decreased Pierce’s disease development [25,163]. Screening of grapevine genes by expression in tomato roots and subsequent cell death assays successfully identified two genes which prevented programmed cell death as a part of Pierce’s disease symptom development. Overexpression of these genes resulted in reduced plant necrosis and *X. fastidiosa* growth [164]. Expression of a polygalacturonase-inhibiting protein (PGIP) in grapevine results in decreased bacterial movement by inhibiting the bacteria’s polygalacturonase [165,166,167].

Transgenic strategies are also being developed for the control of *C.* Las. Citrus expressing the *Arabidopsis* defense gene NPR1 is more tolerant to *C.* Las [168]. Transgenic citrus expressing defensin genes from spinach has shown increased tolerance to *C.* Las [169]. Evaluation of citrus varieties for *C.* Las tolerance and QTL mapping for quantitative tolerance provides background information for development and deployment of transgenics [170,171]. In addition, an asymptomatic *Citrus tristeza virus* strain can be used to deliver therapeutics directly into citrus phloem [172,173], including RNA interference signals that are ingested by *D. citri* and interfere with psyllid development. Although transgenic citrus has not been widely deployed for a myriad of reasons, transgenic tools may ultimately be an important component of a long-term and durable HLB control strategy. 

## 5. Conclusions

Newly emerging plant disease epidemics begin without identification of the causative agent, knowledge of its mode of infection and dispersal, or established tools to study and cultivate the pathogen. Growers and scientists must respond to a crisis scenario quickly, thoughtfully and with a highly coordinated, interdisciplinary effort. In the case of Pierce’s disease, a rapid coordinated response contained the spread of *X. fastidiosa* in California. Scientists made rapid progress by culturing the bacteria and developing mutagenesis protocols. Mutant screening experiments revealed genes required for *X. fastidiosa* virulence, biofilm formation, and transmissibility. This information has been used to create transgenic plants more tolerant of *X. fastidiosa* infection [86]. While *C.* Las has not been cultured, high throughput OMICs techniques and the use of *Liberibacter crescens* as a related culturable model can provide the candidate genes needed to mirror the strategy used for control of *X. fastidiosa.* Like *X. fastidiosa, C.* Las began unidentified and unculturable, yet has specific cues to induce biofilm formation and pathogenesis of both the plant and insect. While the citrus industry is still in a crisis stage for HLB, scientists can reflect on the successful strategies used for Pierce’s disease to mitigate the disaster while looking ahead for more durable HLB control (Table 4). Since OMICs experiments alone may not result in the clear answers that can be gained from mutagenesis experiments, efforts to circumvent the limitation of the unculturability of *C*. Las are paramount, as are the development of strategies that block *C*. Las transmission.

The differences between *C.* Las and *X. fastidiosa* are as informative as their similarities. *X. fastidiosa* forms biofilms in both the plant and insect host, and can both synthesize and perceive biofilm-inducing signals. *X. fastidiosa* grows best in media based on the composition of xylem sap, with consideration for the amino acids that the bacteria can synthesize independently [179]. This successful strategy supports the hypothesis that the environment supporting biofilm formation also contains the necessary substrates for bacterial growth. *C.* Las has not been observed to form biofilms in its plant host, but is thought to form biofilms in *D. citri*, potentially in response to signals from *D. citri* or its bacterial endosymbionts. Efforts to culture *C.* Las have focused on citrus juice and phloem exudate, but have been unsuccessful at establishing propagative bacterial growth [77,78,79]. Following the hypothesis that the biofilm-supporting host is the ‘natural,’ more extensively coevolved host, further efforts should focus on mimicking the environment of *D. citri* rather than that of the plant to establish *C*. Las in culture. Concordant with this recommendation, pathogenic *Liberibacter* bacteria are thought to have diverged and coevolved with their insect vectors millions of years ago [9]. *C.* Las is found in every *D. citri* organ, replicates in the insect, and generally has neutral to beneficial impacts on *D. citri* [135,180]. These facts, in addition to the fact that no canonical resistance has been found in any citrus species suggests that *C.* Las shares a longer evolutionary relationship with *D. citri* than with citrus. Thus, studies of the relationship between bacteria and insect hold the most promise for *C.* Las culture and resistance. 

## Figures and Tables

**Table 1:** Comparison of *Xylella fastidiosa* and *Candidatus* Liberibacter asiaticus traits.

*Xylella fastidiosa*	*Candidatus* Liberibacter asiaticus
Pierce’s disease was first thought to be caused by a virus.	HLB was first thought to be caused by a virus.
Xylem-limited	Phloem-limited
Gammaprotobacteria (includes other Xanthomonads)	Alphaprotobacteria (includes *Rickettsia, Agrobacterium, Rhizobium, Wolbachia*)
Transmitted by hemipteran insect	Transmitted by hemipteran insect
Lacks a type III secretion system	Lacks a type III secretion system
Genome may contain four predicted prophages	Genome may contain prophage
Forms biofilms in insect, plant, and *in vitro*	Forms biofilms in insect, not observed in plant
Culturable	Non-culturable
Propagative, foregut-borne transmission	Propagative, circulative transmission
Generalist pathogen, in which addition of a small number of genes or plasmids can alter host specificity	High level of host and vector specificity

**Table 2:** Selected OMICs resources.

OMIC Resource	Bacteria	Insect Vector	Plant
Genome	*X. fastidiosa* genome, CVC strain 9a5c [30] *X. fastidiosa* genome, Pierce’s disease strain Temecula1 and comparative genomics analyses [31] *X. fastidiosa* biocontrol strain EB92 genome and comparative genomics analyses [32] Comparative genome analysis of 72 *X. fastidiosa* genomes, with 36 newly sequenced genomes presented [33]	GWSS genome [34]	Draft genome of *Vitis vinifera* [35]
	*C.* Las genome [36,37,38,39] *C.* Lam genome [40] *C.* Laf genome [41] *C.* Lso genome [42] *L. crescens* genome [43] Comparative genomics among Liberibacter species and relatives [36,43,44,45,46]	*D. citri* genome [47,48,49] Metagenomics analysis of infected citrus phloem [50]	Draft genome of *Citrus sinensis* [51]
Transcriptome	*X. fastidiosa* transcriptome, CVC strain 9a5c [52,53] and J1a12 [54]	GWSS *de novo* transcriptome and mRNA profile [55,56]	Infected grapevine transcriptome [57]
	Transcriptome not available due to culture challenges	*D. citri* whole-body, antenna, abdominal, gut transcriptome [58,59,60]	Comparative transcriptome of infected rough lemon and sweet orange [61] Microarray comparison of healthy and infected sweet orange [62]
Proteome	*X. fastidiosa* proteome, CVC strain 9a5c [63] *X. fastidiosa* biofilm proteome, CVC strain 9a5c [64]	Not available	Infected grapevine proteome [57] Comparison of proteomes in infected and healthy grapevine [65] Proteomic comparison of tolerant and susceptible grapevine [66]
	Proteome not available due to culture challenges	*D. citri* whole-body, gut, and hemolymph proteome [59,67,68]	Citrus fruit proteome [69] Proteomic analysis of infected pre-symptomatic and symptomatic grapefruit (*Citrus paradisi*) [70]
Metabolome	Metabolome not available	Not available	Infected grapevine metabolome [57] Metabolomics response of olive trees to *X. fastidiosa* subsp. *pauca* [71]
	Metabolome not available due to culture challenges	*D. citri* hemolymph metabolome [72] Metabolic comparison of infected and healthy nymphs [73]	Metabolic comparison of phloem sap from *Murraya paniculata*, *Citrus sinensis*, and *Bergera koenegii* [74] Metabolic analysis of citrus leaves infected or uninfected with *C.* Las, fed on by healthy *D. citri* [75] Metabolic comparison of juice from healthy and infected *Citrus sinensis* [76]

**Table 3:** Description of bacterial genes referenced in texts.

Gene Name	Function	Relevant Bacterium	Importance
*tolC*	Outer membrane component of type I secretion system	*X. fastidiosa*	Knockout causes avirulence and hypersensitivity to phytoalexins
*rpf* gene cluster	Diffusible signal factor (*Xf*DSF) synthesis and recognition	*X. fastidiosa*	Expression in grapevine reduces *X. fastidiosa* spread
*lesA*	Lipase/esterase	*X. fastidiosa*	Key pathogenicity factor for *X. fastidiosa*
*pilA2* & *pilC*	Type 4 pili proteins	*X. fastidiosa*	Involved in biofilm formation
*xadA1 & xadA2*	Afimbrial adhesins	*X. fastidiosa*	Involved in biofilm formation
*mopB*	*X. fastidiosa* outer membrane protein	*X. fastidiosa*	Deletion affects biofilm formation and virulence, eliminates twitching motility
*luxI*	Encodes enzymes that produce acyl-homoserine lactone (AHL) molecules	*C.* Las	*C.* Las lacks a *luxI* gene
*luxR*	AHL-responsive regulatory gene	*C.* Las	*C.* Las possesses a *luxR* gene
*hxfA*	Hemagglutinin-like	*X. fastidiosa*	Deletion results in hypervirulence; plants expressing the gene had decreased disease development
*lasAI & lasAII*	Type V autotransporters	*C.* Las	Found in *C.* Las genome; may target plant mitochondria

**Table 4:** Summary and proposed strategies for HLB research.

Challenge	Pierce’s Disease	HLB	Proposed Strategy for HLB Field
Pathogen culturability	Pathogen can be cultured	Culture is currently not possible	Leverage ‘omic data from *D. citri* to replicate nutritional environment from insect for *C.* Las growth; *in lieu* of culture, test candidate gene functions using *L. crescens*, delivery of RNA and other inhibitory molecules [130,131]
Presence of insect vector	Management via monitoring of nursery stocks; scouting for GWSS; biological control of GWSS using parasitic wasps [150]; eradication has been achieved in specific areas of California [150]	Management via monitoring of nursery stocks; scouting for *D. citri*; early detection of infected trees; biological control of *D. citri* using parasitic wasps [151,152,153] and entomopathogenic fungi [156,157]	Continued aggressive scouting for *D. citri*; test *D. citri* nymphs via PCR for detection of early *C.* Las infection; target plant sampling to sites of *D. citri* feeding by monitoring stylet sheath deposition [174]; evaluate use of *D. citri-*infecting viruses and fungi for wide-scale use; apply control strategies in holistic manner with consideration for potential interactions between biological control agents [158]
Bacterial biofilm formation	Forms biofilm in both insect and plant [98]; adhesion proteins play a role in biofilm formation [103]; outer membrane protein MopB is important for biofilm formation, systemic colonization of xylem [104,105]	Bacterium has a *luxR* but not a *luxI* gene; reported to form biolfilm in insect but not plant [108,109,110]; interaction with other bacterial species may facilitate biofilm formation [58,112,113]	Evaluate importance of outer membrane proteins for biofilm formation; investigate interactions between *C.* Las and *D. citri* endosymbionts; *L. crescens* as a model to study biofilm formation [96]
Transmission by insect vector	Paratransgenesis shows promise to reduce bacterial titer in insect foregut [116]; hemagglutinin and adhesion proteins are involved in transmission [132]; lectins and N-acetylglucosamine reduce transmission [101] White kaolin increases GWSS mortality [136,137]	Color morphology impacts vectoring capacity [133]; vector competency varies naturally among *D. citri* populations	Evaluation of *Wolbachia* repressor protein to control *C.* Las [113]; evaluate induction of *C.* Las prophage; further research into the molecular basis for vector manipulation; delivery of molecules that inhibit feeding structures of *D. citri;* mine secreted salivary proteome for target proteins with potential roles in insect feeding [148]
Infection of host plant	Harpin reduces disease incidence [137]; several conventionally bred and transgenic plants show increased resistance [161,175]; asymptomatic strain of *X. fastidiosa* as biological control	Transgenic citrus shows increased tolerance to *C.* Las [168]; studies of citrus varieties’ tolerance provides resources for breeding and engineering [170,171]; viral-based vector systems allow therapeutics to be delivered into trees [172,173]	Evaluation of *L. crescens* as biological control agent; induction of *C.* Las phages [117,118,119]; use of delivery systems to deliver RNA and other therapeutics into trees based on OMIC and other functional studies; traditional breeding based on tolerance information; generation of transgenic lines expected to have increased tolerance
Climate change	Changing climate resulted in expanded range of GWSS; severity of Pierce’s disease is negatively associated with severity of winter [120,121]; warming climate is expected to expand distribution of pathogen and insect vector	*C.* Las is very heat tolerant relative to other pathogenic Liberibacter species [124]; rising temperature may expand the range of *C.* Las and *D. citri*, and could allow *C.* Las to outcompete other Liberibacters associated with HLB disease	Monitor geographical range of *D. citri* and apply predictive models to anticipate spread of the vector and pathogen over time; adapt existing mathematical models to predict effects of extreme weather events in a strategy analogous to that used for human epidemiology [176]
Non-biological factors	Regulation and certification of nursery stock and bulk grape material can prevent spread the GWSS and/or *X. fastidiosa*; changes in trade agreements among nations may force countries to look to new markets for these products, bringing with them different strains and isolates [177]	Movement of plant material can contribute to spread of *D. citri* and/or *C.* Las; disease spread has been observed to follow truck routes in California [178]; changes in trade agreements among nations may force countries to look to new markets for these products, bringing with them different strains and isolates [177]	Continued quarantine and regulation of citrus material to prevent spread of *D. citri* and *C.* Las; regulation of routes of transport; engagement with legislators to reduce inadvertent disease spread via new trade relationships; development of an international framework for enhanced collaboration among afflicted countries, including sharing information about pathogen detection and disease management strategies
